# A cross-sectional study on gaming intensity and social vulnerability in adolescents that have a chronic condition

**DOI:** 10.3389/fpubh.2023.1128156

**Published:** 2023-04-17

**Authors:** Dionysis Alexandridis, Sanne L. Nijhof, Vincent G. van der Rijst, Damian Y. van der Neut, Renske Spijkerman, Gonneke W. J. M. Stevens, Sander C. J. Bakkes, Heidi M. B. Lesscher, Regina J. J. M. van den Eijnden, Cornelis K. van der Ent, Gerrit van den Berg, Margot Peeters

**Affiliations:** ^1^Multimedia Group, Interaction Division, Department of Computer Science, Faculty of Science, Utrecht University, Utrecht, Netherlands; ^2^Department of Paediatrics, Wilhelmina Children's Hospital, University Medical Center Utrecht, Utrecht University, Utrecht, Netherlands; ^3^Department of Interdisciplinary Social Science, Faculty of Social and Behavioural Sciences, Utrecht University, Utrecht, Netherlands; ^4^Parnassia Addiction Research Centre (PARC, Brijder Addiction Treatment), The Hague, South Holland, Netherlands; ^5^Department of Population Health Sciences, Division of Behavioural Neuroscience, Faculty of Veterinary Medicine, Utrecht University, Utrecht, Netherlands

**Keywords:** chronic condition, children, gaming, problematic gaming, social vulnerability

## Abstract

**Background:**

Adolescents growing up with a chronic condition might experience more social vulnerabilities compared to their healthy peers as an indirect result of their conditions. This can lead to a relatedness need frustration for these adolescents. Consequently, they might spend more time playing video games compared to their peers. Research shows that both social vulnerability and gaming intensity are predictors for problematic gaming. Therefore, we investigated if social vulnerability and gaming intensity are more pronounced in adolescents that have a chronic condition compared to the general population; and if these levels reflect the levels of a clinical group being treated for Internet Gaming Disorder (IGD).

**Methods:**

Data on peer problems and gaming intensity were compared from three separate samples: a national representative sample of adolescents, a clinical sample of adolescents that are undergoing treatment for IGD, and a sample of adolescents diagnosed with a chronic condition.

**Results:**

No differences were found on either peer problems or gaming intensity between the group of adolescents that have chronic conditions and the national representative group. The group with chronic conditions scored significantly lower on gaming intensity than the clinical group. No significant differences were found between these groups on peer problems. We repeated the analyses for boys only. Similar results were found for the group with chronic conditions compared to the national representative group. The group with chronic conditions now scored significantly lower on both peer problems and gaming intensity than the clinical group.

**Conclusion:**

Adolescents growing up with a chronic condition appear similar in their gaming intensity and peer problems compared to their healthy peers.

## 1. Introduction

Video games have become a mainstream source of entertainment and leisure time for youth and adults. According to national prevalence estimates, respectively, 75 and 89% of adolescents in primary (4–12 year old) and secondary (12–16 year old) education play (online) video games in the Netherlands (1). In the Netherlands, 8–14 year olds play on average more than one hour per day with video games (2). Socially vulnerable individuals (i.e., who experience difficulties in their social relationships) spend more hours playing video games than their peers (3) as they are more likely to find reassurance in playing video games (4). Social vulnerability and higher gaming intensity have previously been associated with problematic gaming (i.e., when gaming negatively impacts daily life); specifically people with social vulnerabilities such as social phobia, problems with forming and maintaining friendships, diminished social competence, increased feelings of loneliness, and lower social competence and empathy, were found to be at an increased risk for developing symptoms of Internet Gaming Disorder (IGD) (4–7). Children and adolescents growing up with a chronic disease like autoimmune diseases, childhood cancer or cystic fibrosis, possibly also spend more time playing video games than their peers. As an indirect result of their disease, they might experience difficulties in their social relations (8–11). Synthesizing the above, it is conceivable that children with a chronic condition play more video games than their healthy peers, increasing their risk on problematic gaming.

Children and adolescents that grow up with a chronic condition are more likely to cope with social vulnerabilities as an indirect results of their condition. They have significantly higher levels of school absenteeism than their peers, attend special forms of educations such as home schooling more often, participate less in public sport clubs, and face more often social challenges such as stigmatization and bullying (12–17). This leads to limited time spent on peer-related activities which might result in impaired social functioning (8–10). Indeed, children with chronic diseases were found to have on average fewer friends, to have more difficulties maintaining friendships (18, 19), and to experience on average more feelings of loneliness than their peers (9). At the same time, they value friendships and feeling accepted as the most important factors in their lives (20, 21). Especially during early adolescence, friendships and peer influence become more prominent (22–24). Specifically between ages 10–14, adolescents are more susceptible to the influence of peers (25–27). Peer pressure, concerns about social rejection, and the desire to be popular have a major influence on adolescents' behavior (26, 28).

From a Self-Determination Theory (SDT) perspective, a theory largely based on motivation and need satisfaction, children and adolescents growing up with a chronic disease are at an increased risk to experience deficiencies in their need satisfaction of relatedness. The *basic psychological needs theory* (BPNT), an important element within the SDT, postulates three universal basic psychological needs that are essential for a person's well-being and psychological growth: *competence*—the feeling of being in control of the outcome of your own actions, *relatedness* — a sense of reciprocal care, value, and belonging in relation to others, and *autonomy*—a sense that actions are self-endorsed and performed willingly (29). Whereas satisfaction of these three basic needs lead to wellbeing, frustration of these needs impairs this. As children and adolescents with a chronic disease are likely to experience a need frustration in their need for relatedness within the real world, they might search for alternatives such as via video games.

Indeed, video games can arguably serve the psychological need for relatedness (30, 31). Video games have the ability to create new (online) relationships that could potentially simulate feelings of belonging and increase perceived social competence (32, 33). Perhaps the most straightforward example of video games that can achieve this are online multiplayer video games in which several to thousands of people play together in large digital worlds. These video games have the potential to create online friendships, and to provide players with a virtual community (e.g., through complex social structures such as “guilds” or “clans”) and a sense of belonging (4, 32, 33). Power and status attainment in video games may also offer a greater reward for socially vulnerable youngsters as they experience this less often in the real world than their more socially competent peers (33).

Whilst these virtual relationships are maintained with other human players, even single player video games provide a solitary player with meaningful and satisfying experiences; for example, through a high sense of immersion and employing narratives in which players must actively engage (34–36). Tyack and Wyeth (31) argue that single player video games can satisfy the need for relatedness through various pathways, including *parasocial relationships* (one-sided relationships with media personas). Indeed, a recent study revealed how players can develop emotional attachments to in-game characters that correspond with themes such as “*concern for one's protege*,” “*trusted close friend*,” or having a “*crush*” (37). It shows that both single player and multiplayer video games have the potential to fulfill the need of relatedness.

Here arises an interesting causation: the *need density hypothesis* argues that when there is a large discrepancy between real-world and in-game need satisfaction, the risk to develop symptoms of pathological gaming will be strongest (38). This has been confirmed in several qualitative and quantitative studies (39–41). Games have three important characteristics with regard to the BPNT: they offer *immediate* access to need satisfaction as they are readily available; they are *consistent* in the satisfaction that is gained as a result of their reliable and predictable rules; and they provide a high *density* in need satisfaction as a result of the many short-term and long-term goals that are present in the game. Video games can be used as a coping mechanism to escape from an unsatisfying life. Coping motivations for video game play have previously been associated with higher problematic gaming (42, 43). In addition, intense gaming has also been associated with an increased risk for problematic gaming (3).

Problematic gaming is associated with a range of negative health outcomes and negative life consequences, such as poorer self-reported school performance, increased feelings of loneliness, increased emotional and behavioral problems, sleep deprivation, social isolation, and poorer psychological wellbeing (2, 4, 7, 44–46). Adolescents with a chronic condition are already at risk for developing psychosomatic problems (10, 11, 47–50). Therefore, it is important to gain a better understanding of whether adolescents with a chronic condition are also more vulnerable for developing symptoms of problematic gaming due to increased social vulnerabilities as a result from their conditions.

Thus far, we have hypothesized that adolescents with a chronic condition generally cope with social vulnerabilities. This can lead to an increase in the hours spent on playing video games. As discussed previously, according the literature social vulnerabilities and intense gaming are predictors for developing symptoms of problematic gaming (3, 6). Consequently, we investigate possible pronounced effects of social vulnerability and gaming intensity among adolescents with a chronic condition by examining data from three different datasets: a sample of adolescents with a chronic condition, a national representative sample, and a clinical sample of adolescents who are undergoing treatment for IGD. First we will establish whether the suggested relationship between peer problems and (problematic) gaming is present in the current national representative sample. Next, we investigate if social vulnerability and gaming intensity are more pronounced in adolescents with a chronic condition compared to the general population, or rather if they reflect the levels of the clinical group.

## 2. Methodology

### 2.1. Procedure

The current study utilizes three separate samples consisting of a national representative sample of Dutch youth, a clinical sample of adolescents in addiction care—seeking help for gaming problems—and a sample that includes adolescents with a chronic condition.

The national representative sample was part of the Health Behavior in School-aged Children (HBSC) study (51). This is an ongoing cross-sectional cohort study collecting data every four years to monitor health and well-being in Dutch adolescents. The current study used the 2017 assessment (*N* = 4,805), that consisted of adolescent gamers aged 12- to 16-years old (M_age_ = 13.68, SD_age_ = 1.36).

The clinical sample data were obtained from a retrospective cohort study based on patient registry data from adolescent patients (12–22 years) receiving addiction treatment at Brijder, a Dutch addiction care facility. The sample (*N* = 89) includes patients aged 12–16 years old (M_age_ = 14.51, SD_age_ = 1.21) with a primary diagnosis of gaming addiction.

Lastly, data for the sample of adolescents that have a chronic condition were obtained from PROactive, a prospective cohort study among pediatric patients who suffer(ed) from a chronic condition or life-threatening conditions such as cystic fibrosis, juvenile idiopathic arthritis, chronic kidney disease, congenital heart disease, inflammatory bowel diseases, systemic auto-immune diseases, or primary immune deficiencies (52, 53). The current study utilizes a subsample consisting of gamers (*N* = 114) aged 12- to 15-years old (M_age_ = 13.84, SD_age_ = 1.47).

Adolescents from each dataset were only included in the present study only if they indicated that they played video games in the last three months.

### 2.2. Measures

The main outcomes were gaming intensity and peer problems. Using the HBSC-study as a baseline, the Brijder and PROactive measures were recoded to allow for a more accurate comparison between samples. For the HBSC and Brijder samples we also include measures for problematic gaming. These were not available for the PROactive sample.

#### 2.2.1. Gaming intensity

For the HBSC and PROactive samples gaming intensity consists of two items; one for measuring how many days a week an individual played games and another measuring the hours spent gaming on a given day. These categories were then recoded in continuous values and multiplied together to calculate an estimation of hours spent playing video games on a weekly basis, with higher scores indicating more hours spent gaming. For the Brijder sample gaming intensity also consists of two items; one for measuring how many days an individual played games in the past 30 days and another measuring the hours spent on a given day (both variables were measured in continuous values). To make this measure consistent to the HBSC and PROactive assessment, the values form the Brijder sample were recoded. First, the amount of days a week an individual played games was recorded by calculating the corresponding weekly frequency in relation to the monthly frequency report (30 days divided by 7 days a week ≈ 4.28). Then the number of days an individual played games in the last 30 days was divided by 4.28, rounding to complete days, to get an estimate of the number of days a week an individual played games. These outcomes were then recoded and multiplied in the same way as was done for the HBSC and PROactive samples to calculate an estimation of hours spent gaming on a weekly basis.

#### 2.2.2. Peer problems

In the present study, peer problems are operationalized by the peer problems sub-scale of the Dutch version of the Strength-Difficulties Questionnaire (SDQ) (54–56). This scale was used in each of the samples. The scale consists of five statements that measure to what extent an individual experiences difficulties with peers, for example “Other children bully me”. All items are answered on a three-point Likert scale (0 = “not true”; 1 = “slightly true”; 2 = “very true”). After recoding two items, the sum of the five items was calculated, were a higher score indicated that an individual experienced more peer problems. Given the ordinal nature of the items, internal consistency was calculated using the ordinal alpha that is based on the polychoric correlation matrix (57). The internal consistency for the peer problems scale is low to medium (ordinal alpha = 0.64), however the scale is validated in a national Dutch sample, being invariant over time and valid between different subgroups (58).

#### 2.2.3. Problematic gaming

In the HBSC and Brijder data, problematic gaming was measured using the Dutch adaptation of the Internet Gaming Disorder scale. Although both samples used measures of IGD, we refer to the outcomes as problematic gaming. We specifically use the terminology “*problematic gaming*” because we are not using a cut-off criterion. Using the IGD scale as a continuous measure, we hope to get a better understanding on the level of problematic gaming behavior rather than an actual diagnosis based on the IGD criteria. HBSC used the Lemmens scale (59), while Brijder used a translation of the DSM-5 items to measure problematic gaming. The scales include nine items corresponding to the nine diagnostic criteria for Internet Gaming Disorder according to the DSM-5. Participants were asked “During the past year, have you (...)”, followed by for example “regularly had no interest in hobbies or other activities because you would rather play video games?”, to which they replied on a dichotomous scale (0 = “no” and 1 = “yes”). The sum of the nine items was calculated, where a higher score indicated having more Internet Gaming Disorder symptoms. Giving the dichotomous nature of the items, internal consistency was calculated using the ordinal alpha that is based on the polychoric correlation matrix (57). Ordinal alpha value was 0.91. The PROactive sample did not include this measure.

### 2.3. Analyzing strategy

First, descriptive statistics and Pearson correlations were provided for each subsample separately. To investigate if peer problems are indeed a vulnerability marker for gaming intensity and/or problematic gaming, we performed a regression analyses for the national representative group examining the relationship between peer problems and gaming intensity, controlling for sex differences. In a second regression, we investigated the relationship between peer problems and IGD symptoms, controlling for sex differences and gaming intensity.

Next, we performed Anova group comparisons using Tukey *post-hoc* tests to observe possible differences on the study variables “gaming intensity” and “peer problems,” and additionally to observe possible differences in sex, age, and IGD symptoms (Brijder and HBSC only). Finally, multinominal logistic regression analyses were performed to analyze the extent to which gaming intensity, peer problems, or sex are predictors of group membership (i.e., Brijder, PROactive, HBSC). Sample was the grouping variable (PROactive as reference category, and in a second step HBSC as reference category), and gaming, sex and peer problems were added as predictors. Since the Brijder sample included almost only males (95%), multinominal regression analyses were repeated for boys only. All analyses were performed using IBM SPSS statistics version 28.

## 3. Results

Mean age was 13.68, 14.51, and 13.84 for the HBSC, Brijder and PROactive groups, respectively. The percentage of boys included in the group was fairly balanced for HBSC and PROactive (63% and 53% respectively), while Brijder included mainly boys (95%). Average gaming intensity was higher for the Brijder group (25.92) as compared to HBSC and PROactive (10.58 and 10.35, respectively). Finally, peer problems were lowest for the PROactive group (1.54), HBSC was a bit higher (1.71), while Brijder had the highest score on peer problems (2.34). See Table 1 for an overview of the descriptive statistics.

**Table 1:** Descriptive statistics of the PROactive, HBSC, and Brijder datasets.

**Full Sample ( *N* = 5008)**	**HBSC ( *N* = 4805)**	**Brijder ( *N* = 89)**	**PROactive ( *N* = 114)**
	**M (SD)**	**M (SD)**	**M (SD)**
Age	13.68 (1.36)	14.51 (1.21)	13.84 (1.47)
Gaming Intensity	10.58^a^ (10.57)	25.92^b^ (14.93)	10.35^a^ (8.88)
Peer problems	1.71^a^ (1.64)	2.34^b^ (1.81)	1.54^a^ (1.80)
% Boy	64%	95%	53%
IGD sum	1.25^a^ (1.74)	4.77^b^ (2.38)	

^a^^b^ Different subscripts letters indicate significant differences between groups. For example, a significant difference was found on gaming intensity between the HBSC sample (superscript “a”) and the Brijder sample (superscript “b”), and between the PROactive sample (superscript “a”) and the Brijder sample (superscript “b”). “Age” represents the mean age of the included participants. “Gaming intensity” indicates the mean number of hours spent playing video games per week. “Peer problems” refers to the mean peer problems score from the SDQ scale. “% Boy” describes the percentage of participants that are a boy. “IGD sum” indicates the average IDG score.

Table 2 presents the Pearson's Correlation Coefficients for age, gaming intensity, peer problems, sex, and IGD for each group. Within the HBSC group, we found medium to strong positive correlates between gaming intensity and peer problems, gaming intensity and IGD, and peer problems and IGD. Negative correlations were found for gaming intensity and sex, indicating that boys play more games than girls. Within the Brijder sample, only a weak negative correlation was found between age and IGD, indicating that younger participants reported more IGD symptoms. Finally, a medium negative correlation was found between gaming intensity and sex for the PROactive sample, indicating that boys play more games than girls.

**Table 2:** Pearson's correlation coefficients for age, gaming intensity, peer problems, sex, and IGD.

**Full sample (*N* = 5,008)**	**HBSC (*****N*** = **4,835)**					**Brijder (*****N*** = **89)**					**PROactive (*****N*** = **114)**			
	1.	2.	3.	4.	5.	1.	2.	3.	4.	5.	1.	2.	3.	4.
1. Age	0.11	−0.19	0.01	−0.07^**^	0.02	1	0.03	0.03	0.04	−0.21^*^	1	0.05	−0.04	−0.04
2. Gaming intensity		1	0.15^**^	−0.32^**^	0.46^**^		1	0.02	0.03	0.18		1	0.07	−0.47^**^
3. Peer problems			1	0.01	0.23^**^			1	0.14	−0.04			1	0.11
4. Boy				1	−0.02				1	0.08				1
5. IGD sum					1					1				

IGD scores were not available for the PROactive group, and therefore not displayed. ^*^p < 0.05; ^**^p < 0.01.

### 3.1. Regression analyses

As a first step, we performed regression analyses between peer problems and gaming intensity. Results revealed a significant positive relationship between peer problems and gaming intensity indicating that more peer problems were associated with more intense gaming behavior in the HBSC sample, while controlling for sex differences (see Table 3). A positive significant association was also found for peer problems and IGD symptoms, indicating that more peer problems were associated with more reported IGD symptoms. This association remained after controlling for possible sex differences and gaming intensity (see Table 4), indicating that beyond hours spend on gaming, peer problems were also associated with problematic symptoms of gaming behavior such as lost of interest in other hobbies and conflict with peers and parents about gaming behavior.

**Table 3:** Regression analysis in the HBSC sample of peer problems on gaming intensity, controlled for sex.

	**Unstandardized coefficients**		**Standardized coefficients**		
	**B**	**Std. error**	**beta**	***t***	**sig**
(Constant)	18.701	0.460		40.696	<0.000
Boy	−7.226	0.302	-0.323	−23.949	<0.001
Peer problems	1.013	0.088	0.155	11.490	<0.001

The dependent variable in the analysis was gaming intensity.

**Table 4:** Regression analysis in the HBSC sample for peer problems on reported IGD symptoms, controlled for gaming intensity and sex.

	**Unstandardized coefficients**		**Standardized coefficients**		
	**B**	**Std. error**	**beta**	***t***	**sig**
(Constant)	1.123	0.079		14.142	<0.001
Boy	–0.613	0.048	–0.169	–12.884	<0.001
Peer problems	0.190	0.013	0.179	14.266	<0.001
Gaming intensity	0.061	0.002	0.375	28.236	<0.001

The dependent variable was the sum-score of the IGD items (the number of times that a person answered yes.

In a second step, we performed multinominal logistic regression analyses to examine the extent to which gaming intensity, peer problems and sex predict group membership (HBSC, Brijder or PROactive). Table 5 presents the results of these analyses. Results indicate that when compared to the national representative group (HBSC), the group with chronic conditions (PROactive) only differed significantly on sex (OR = 0.59; 95% CI 0.40–0.88) indicating that males were less likely to be in the group that have chronic conditions. A marginal difference was found on gaming intensity between between the group with chronic conditions and the national representative sample (OR = 0.99; 95% CI 0.97–1.01). When compared to the clinical group (Brijder), the group with chronic conditions differed significantly on sex (OR = 0.09; 95% CI 0.03–0.26) as well as on gaming intensity (OR = 1.05; 95% CI 1.03–1.08). These results indicate that group membership for the group with chronic conditions (when compared to the clinical group) was associated with lower scores on gaming intensity and less likely to be male.

**Table 5:** Multinominal regression analyses with subsample as grouping variable, and sex, gaming and peer problems as predictors.

	**National representative (HBSC)**				**Clinical group (Brijder)**			
	***N*** = **4,835**				***N*** = **89**			
	**OR**	**B**	***p***	**95% CFI**	**OR**	**B**	***p***	**95% CFI**
Sex	0.59	–0.53	0.42	0.40–0.88	0.09	–2.45	<0.01	0.03–0.26
Gaming intensity	0.99	–0.01	0.01^*a*^	0.97–1.01	1.05	0.05	<.01	1.03–1.08
Peer problems	1.08	0.07	0.24	0.95–1.21	1.18	0.17	0.05	0.99–1.40

^a^*p* <0.05. Reference group is the group with chronic conditions (PROactive, *N* = 114).

Since males were over-represented in the clinical group, the multinominal logistic regression analyses were repeated for males only (see Table 6). Results illustrated that boys in the group with chronic conditions (PROactive) when compared to the national representative group (HBSC) did not significantly differ on peer problems or gaming intensity, indicating that boys in both groups report similar levels of peer problems and gaming intensity. When compared to the clinical group (Brijder), boys in the group with chronic conditions scored significantly lower on gaming intensity (OR = 1.05; 95% CI 1.02–1.08) and peer problems (OR = 1.27; 95% CI 1.03–1.58), indicating that group membership to the group with chronic conditions (when compared to the clinical group) was associated with lower scores on gaming intensity and peer problems.

**Table 6:** Multinominal regression analyses with subsample as grouping variable, and gaming and peer problems as predictors for boys only.

	**National representative (HBSC)**				**Clinical group (Brijder)**			
	***N*** = **3,090**				***N*** = **85**			
	**OR**	**B**	***p***	**95% CI**	**OR**	**B**	***p***	**95% CI**
Gaming Intensity	0.98	–0.1	0.30	0.97–1.01	1.05	0.05	<0.01	1.02–1.08
Peer Problems	1.17	0.16	0.08	0.98–1.41	1.27	0.24	0.03	1.03–1.58

Reference group is the group with chronic conditions (PROactive, *N* = 60).

In a third step we compared the national representative group (HBSC) to the clinical group (Brijder). Results revealed a significant difference for sex (OR = 0.15; 95% CI 0.05–0.40) and gaming intensity (OR = 1.06; 95% CI 1.05–1.08), indicating that group membership for the national representative sample (when compared to the clinical group) was associated with lower scores on gaming intensity and more likely being a male.

The national representative group (HBSC) only including boys was also compared to the clinical group (Brijder) revealing significant differences for gaming intensity (OR = 1.06; 95% CI 1.05–1.08, not presented in the tables), indicating that group membership to the national representative sample is associated with lower scores on gaming intensity.

## 4. Discussion

The purpose of the present study was to explore if vulnerabilities for developing problematic gaming behavior are more pronounced in adolescents that have a chronic condition. To this end we investigated if levels of social vulnerability (operationalized as peer problems) and gaming intensity are more pronounced in adolescents with a chronic condition than in the general population; and if these reflect the levels of adolescents undergoing treatment for IGD. Interestingly, the results showed no significant differences between the group with chronic conditions and the general population. Moreover, the group with chronic conditions scored significantly lower than the clinical group on gaming intensity. When only considering boys in both groups, peer problems were significantly lower in the group with chronic conditions compared to the clinical group. Adolescents with a chronic condition do not have more peer problems, nor do they game more hours a week when compared to the general population, suggesting that when considering these vulnerabilities, they are not at an increased risk for developing problematic gaming behavior.

We also examined if peer problems are indeed positively related with gaming intensity and IGD scores to establish if peer problems are a vulnerability marker for increased gaming intensity and problematic gaming behavior. The regressions for peer problems showed a positive relation with gaming intensity, and with IGD scores. Furthermore, positive correlations were found for gaming intensity and peer problems, gaming intensity and IGD scores, and peer problems and IGD scores within the HBSC group. In line with the literature, these results do indeed show that higher peer problems are predictive of a higher gaming intensity, and of higher IGD scores (3, 39–41). These correlations were not found within the clinical group nor in the group with chronic conditions. The adolescents within the clinical group are most likely quite homogeneous in terms of peer problems and gaming intensity. This is to be expected, as IGD is associated with elevated levels of peer problems and gaming intensity. Therefore, it is not surprising that we cannot find any correlations on these measures within such a homogeneous group.

### 4.1. Chronic conditions, peer problems, and gaming

Contrary to our hypothesis, adolescents that have a chronic condition did not score higher on peer problems (mean = 1.54) compared to their healthy peers (mean = 1.71). Berkelbach van der Sprenkel et al. (50) studied the psychosocial functioning of adolescents with a chronic condition compared to their peers, using the HBSC data from 2013. Their results showed that, among other findings, adolescents with a self-reported chronic condition scored on average significantly higher on *peer problems*, and lower on *peer support*. An important difference between our study and theirs is that adolescents included in the PROactive study have been diagnosed with a chronic condition by a clinician, while Berkelbach van der Sprenkel et al. (50) used self-reported measures for having a chronic condition. Interestingly, only 25% of the adolescents in the PROactive study who had a chronic condition self-reported their chronic condition on a questionnaire (data not published). It is possible that adolescents who *perceive* themselves as having a chronic condition, regardless of clinicians agreeing, is the at-risk group for experiencing elevated levels of social vulnerability. Nevertheless, they did not find any significant differences between the self-reported group of having a chronic condition and their healthy peers on gaming intensity.^1^ These results suggest that adolescents growing up with a chronic condition, regardless of the condition being diagnosed by clinicians or being self-reported, are similar in their gaming behavior compared to their healthy peers.

In line with previous research, we found no differences in gaming intensity between the PROactive group and the HBSC group (50). We argued that because video games can be a means to create and maintain friendships, we would expect a (slightly) higher gaming intensity for adolescents that have a chronic condition. Adolescents growing up with a chronic condition report on average higher levels of chronic fatigue, including cognitive fatigue (47). Playing with video games often requires a high cognitive load from the players (60). Perhaps adolescents with a chronic condition are limited in their (desired) gaming intensity by other factors such as chronic fatigue. Further investigation is required to fully understand the gaming intensity of adolescents that have a chronic condition.

### 4.2. Understanding the results via the need-density hypothesis

The need density hypothesis argues that the risk for developing symptoms of pathological gaming is greatest, when there is a large discrepancy between real-world and in-game need satisfaction. We hypothesized that because adolescents with a chronic condition are more often restricted in their social participation (12–17), they would have an increased risk for developing peer problems (8–10, 18, 19). Thus, we expected to see an increase in gaming intensity compared to the general population. However, the results showed similar gaming intensity and peer problems for adolescents that have a chronic condition and the general population.

Adolescents that have a chronic condition in the current sample do not experience elevated levels of peer problems when compared to their healthy peers. Therefore, according to the need density hypothesis, we should not expect to see an increase in gaming intensity for these adolescents; as is reflected by the results. It is possible that other, more subtle social interactions are affected by their health conditions that are not apparent in instruments that are designed to be used for clinical screening (such as the SDQ). As mentioned in Section 1, the basic psychological needs theory (BPNT) is reflected in three important properties of video games: they can offer *immediate, consistent*, and *dense* satisfaction of the basic psychological needs. If the social vulnerabilities associated with chronic conditions affect more subtle social interactions, it could be that the *immediate* and *consistent* properties are the instrumental ones to this group's need satisfaction. In that case, the experience would not require to be *dense* to achieve need satisfaction.

This insight is supported by interviews that were conducted in parallel to the present study at Utrecht University (61). These interviews were set up to gain a deeper understanding of the motivations for playing video games in adolescents with and without having a chronic condition. Although the adolescents that have a chronic condition often mentioned social motives for playing video games, they stated that they employed video games as complementary to their offline social interactions rather than as a replacement. As most of the relatedness need satisfaction is fulfilled in the offline interaction of these adolescents, the density property of video games likely becomes of little significance. Interestingly, the healthy adolescents that were interviewed in this study also indicated similar social motives for playing video games (to complement their offline relationships). This emphasizes the similarity between both groups even further.

## 5. Limitations and future works

In this section we discuss the limitations of the present study and explore some interesting pathways for the future. One limitation of the current study is that the formulation of IGD symptoms in the Brijder and HBSC study are slightly different. Both questionnaires were set up to measure problematic gaming behavior according to the nine DSM-5 items. However, Brijder used a direct translation of the DSM-5 items while HBSC used the Lemmens scale (59), which uses slightly different phrasing to better fit the language use of adolescents. However, since we only compared gaming intensity, and peer problems directly between the groups and not IGD scores, we expect potential biases to be minimal.

Another limitation of the current study is that we had to re-encode the Brijder data on gaming intensity to be able to compare it to the other groups. This could result in a slight distortion of the results. However, since the data were carefully and logically re-encoded to match the other groups, the potential bias will be minimal.

Due to the cross-sectional nature of our study, we cannot identify the direction of the effect between peer problems and gaming intensity, and between peer problems and IGD scores. Peer problems both predict and are affected by problematic game play (6, 44). Nevertheless, whether peer problem differences are a result or a predictor of problematic gaming, peer problems did significantly differ between groups.

It would be interesting to investigate other forms of social vulnerability in future works. Social vulnerability was operationalized by the ‘peer problems' sub-scale from the SDQ. Although peer problems are a form of social vulnerability, it does not represent the entire array of possible social vulnerabilities. It could be that social vulnerabilities experienced by adolescents that have a chronic condition are not captured by the ‘peer problems' scale from the SDQ. Therefore, future studies should include various measures of social vulnerability to gain a better understanding of the vulnerabilities associated with chronic conditions.

Besides including various measures corresponding to social vulnerabilities, it is of importance to also include measures capturing less pronounced social interactions. As mentioned in Section 4.2, the social vulnerabilities associated with chronic conditions could be more subtle. Screening instruments are typically not suitable to capture more nuanced differences in behavior and functioning. It might be that adolescents with a chronic condition experience various more nuanced social vulnerabilities, that together may impact their need for relatedness. It would be interesting to investigate if this hypothesis is indeed true and how this relates to gaming behavior.

In the present study we examined adolescents' gaming behavior only through gaming intensity, as this was the only data on gaming behavior available for each dataset. It would have been more insightful to compare multiple aspects of gaming behavior in future studies. For example, comparing IGD scores directly between groups (including PROactive) offers a better understanding of problematic gaming behaviors. Although gaming intensity is a known predictor for IGD, it could be that gaming intensity is not a predictor of IGD for adolescents that have a chronic condition. As mentioned earlier, there may be other limiting factors for the gaming intensity of adolescents with chronic conditions such as chronic fatigue. As such, an interesting research focus would be the interplay between the person that is playing the game (Who), the motivation and goals for playing (Why), the social context (Where and with Whom), and the type of game interactions that are experienced by the player (What) (62, 63). We agree with Verheijen (63) that the multidimensionality of video game effects needs to be acknowledged, as indeed there is no uniform effect to be expected of video games on well-being. Therefore, for future work, we propose to focus less on gaming density and frequency, and more on what is actually experienced by (distinct) individuals during their play experiences.

## 6. Conclusion

The present study found that peer problems are indeed a vulnerability marker for an increased gaming intensity and problematic gaming in the general population. We also showed that adolescents with a chronic condition are similar in their gaming behavior and peer problems compared to their healthy peers. Furthermore, the present study found that adolescents that have a chronic condition spend significantly less time playing video games and have considerably less peer problems than adolescents undergoing treatment for IGD. These results suggest that adolescents with a chronic condition are not at a greater risk than their healthy peers to develop problematic gaming behavior. Although the present study provides interesting and relevant insights, it is important to consider different types of social vulnerabilities and gaming behavior in future studies, to create a more complete understanding of the potential risks of developing symptoms of problematic gaming that adolescents with a chronic condition might face. Finally, as is the case in the general population, some individuals that have a chronic condition may be more vulnerable for problematic gaming, irrespective of their condition. It is therefore important that clinicians and pediatricians continue to evaluate to what extent their patients feels well connected to their peers and has satisfying friendship networks.

## Data availability statement

The original contributions presented in the study are included in the article/supplementary material, further inquiries can be directed to the corresponding author.

## Author contributions

DA: conceptualization, writing—original draft, and writing—review and editing. SN: supervision, providing the PROactive dataset, and writing—review and editing. VR and DN: preliminary analysis, writing—methodology, and interview study. RS: providing Brijder data set and writing—review. GS and RE: providing HBSC data set and writing—review. SB and HL: writing—review and editing. CE and GB: writing—critical review. MP: formal analysis and writing—review and editing. All authors contributed to the article and approved the submitted version.

## Footnotes

^1^Gaming intensity in (50) is defined as the number of hours a day spend playing video games.

## References

[1] Rombouts M van Dorsselaer S Scheffers van Schayck T Tuithof M Kleinjan M Monshouwer K. Jeugd en riskant gedrag 2019. (Trimbos-instituut) (2020).

[2] van Rooij A Dalinghaus N van den Eijnden R. (on) gezond gamegedrag van nederlandse jongeren. JGZ Tijdschrift voor jeugdgezondheidszorg. (2020) 52:45–50. 10.1007/s12452-020-00211-w

[3] Rosendo-Rios V Trott S Shukla P. Systematic literature review online gaming addiction among children and young adults: a framework and research agenda. Addict Behav. (2022) 129:107238. 10.1016/j.addbeh.2022.107238 35104738

[4] Lemmens JS Valkenburg PM Peter J. Psychosocial causes and consequences of pathological gaming. Comput Hum Behav. (2011) 27:144–52. 10.1016/j.chb.2010.07.015

[5] Sioni SR Burleson MH Bekerian DA. Internet gaming disorder: aocial phobia and identifying with your virtual self. Comput Hum Behav. (2017) 71:11–5. 10.1016/j.chb.2017.01.044

[6] Peeters M Koning I van den Eijnden R. Predicting internet gaming disorder symptoms in young adolescents: a one-year follow-up study. Comput Hum Behav. (2018) 80:255–61. 10.1016/j.chb.2017.11.008

[7] Gentile DA Choo H Liau A Sim T Li D Fung D. Pathological video game use among youths: a two-year longitudinal study. Pediatrics. (2011) 127:e319–29. 10.1542/peds.2010-1353 21242221

[8] Shute RH Walsh C. Adolescents with chronic illnesses: achool absenteeism, perceived peer aggression, and loneliness. Sci World J. (2005) 5:535–44. 10.1100/tsw.2005.68 16075150 PMC5936595

[9] Maes M Van den Noortgate W Fustolo-Gunnink SF Rassart J Luyckx K Goossens L. Loneliness in children and adolescents with chronic physical conditions: a meta-analysis. J Pediatr Psychol. (2017) 42:622–35. 10.1093/jpepsy/jsx046 28340072

[10] Pinquart M Shen Y. Behavior problems in children and adolescents with chronic physical illness: a meta-analysis. J Ppediatr Psychol. (2011) 36:1003–16. 10.1093/jpepsy/jsr042 21810623

[11] Maurice-Stam H Nijhof SL Monninkhof AS Heymans HS Grootenhuis MA. Review about the impact of growing up with a chronic disease showed delays achieving psychosocial milestones. Acta Paediatr. (2019) 108:2157–69. 10.1111/apa.14918 31250466

[12] Emerson ND Distelberg B Morrell HE Williams-Reade J Tapanes D Montgomery S. Quality of life and school absenteeism in children with chronic illness. J Schl Nurs. (2016) 32:258–66. 10.1177/1059840515615401 26572160 PMC4867299

[13] Lum A Wakefield C Donnan B Burns M Fardell J Marshall G. Understanding the school experiences of children and adolescents with serious chronic illness: a systematic meta-review. Child Care Health Dev. (2017) 43:645–62. 10.1111/cch.12475 28543609

[14] Richardson KL Weiss NS Halbach S. Chronic school absenteeism of children with chronic kidney disease. J Pediatr. (2018) 199:267–71. 10.1016/j.jpeds.2018.03.031 29706492 PMC6063782

[15] van Hal L van Rooijen M van der Hoff M. Een Actueel Perspectief op kinderen en jongeren met een chronische aandoening in Nederland: Omvang, Samenstelling en Participatie. Utrecht: Verwey-Jonker Insituut (2019).

[16] McMaugh A. En/countering disablement in school life in australia: Children talk about peer relations and living with illness and disability. Disa Soc. (2011) 26:853–66. 10.1080/09687599.2011.618740

[17] Pinquart M. Systematic review: bullying involvement of children with and without chronic physical illness and/or physical/sensory disability—a meta-analytic comparison with healthy/nondisabled peers. J Pediatr Psychol. (2017) 42:245–59. 10.1093/jpepsy/jsw081 27784727

[18] Forgeron PA King S Stinson JN McGrath PJ MacDonald AJ Chambers CT. Social functioning and peer relationships in children and adolescents with chronic pain: a systematic review. Pain Res Manag. (2010) 15:27–41. 10.1155/2010/820407 20195556 PMC2855293

[19] Woodgate RL Gonzalez M Demczuk L Snow WM Barriage S Kirk S. How do peers promote social inclusion of children with disabilities? a mixed-methods systematic review. Disab Rehabil. (2020) 42:2553–79. 30907279 10.1080/09638288.2018.1561955

[20] Spencer-Cavaliere N Watkinson EJ. Inclusion understood from the perspectives of children with disability. Adapt Phys Activity Quart. (2010) 27:275–93. 20956835 10.1123/apaq.27.4.275

[21] Taylor RM Gibson F Franck LS. The experience of living with a chronic illness during adolescence: a critical review of the literature. J Clin Nurs. (2008) 17:3083–91. 10.1111/j.1365-2702.2008.02629.x 19012778

[22] Brown BB. Adolescents' relationships with peers. In: Handbook of Adolescent Psychology. New York, NY: Wiley (2004). p. 363–94.

[23] de Boer A Peeters M Koning I. An experimental study of risk taking behavior among adolescents: a closer look at peer and sex influences. J Early Adolesc. (2017) 37:1125–41. 10.1177/0272431616648453

[24] Youniss J Smollar J. Adolescent Relations with Mothers, Fathers and Friends. Chicago, IL: University of Chicago Press (1987).

[25] Steinberg L Monahan KC. Age differences in resistance to peer influence. Dev Psychol. (2007) 43:1531. 10.1123/apaq.27.4.275 18020830 PMC2779518

[26] Forbes EE Dahl RE. Pubertal development and behavior: hormonal activation of social and motivational tendencies. Brain Cogn. (2010) 72:66–72. 10.1016/j.bandc.2009.10.007 19942334 PMC3955709

[27] Newcomb AF Bukowski WM Pattee L. Children's peer relations: a meta-analytic review of popular, rejected, neglected, controversial, and average sociometric status. Psychol Bull. (1993) 113:99. 10.1037/0033-2909.113.1.99 8426876

[28] Prinstein MJ Brechwald WA Cohen GL. Susceptibility to peer influence: using a performance-based measure to identify adolescent males at heightened risk for deviant peer socialization. Dev Psychol. (2011) 47:1167. 10.1037/a0023274 21463036 PMC3348704

[29] Deci EL Ryan RM. Self-determination theory: a macrotheory of human motivation, development, and health. Can Psychol. (2008) 149:82. 10.1037/a001280131999139

[30] Przybylski AK Rigby CS Ryan RM. A motivational model of video game engagement. Rev Gen Psychol. (2010) 14:154–66. 10.1037/a0019440

[31] Tyack A Wyeth P. Exploring relatedness in single-player video game play. In: Proceedings of the 29th Australian Conference on Computer-Human Interaction. Brisbane, QLD: Association for Computing Machinery (2017). p. 422–7.

[32] Beranuy M Carbonell X Griffiths MD. A qualitative analysis of online gaming addicts in treatment. Int J Mental Health Addict. (2013) 11:149–61. 10.1007/s11469-012-9405-2

[33] King DL Delfabbro PH. The cognitive psychology of internet gaming disorder. Clin Psychol Rev. (2014) 34:298–308. 10.1016/j.cpr.2014.03.006 24786896

[34] Ryan RM Rigby CS Przybylski A. The motivational pull of video games: a self-determination theory approach. Motiv Emot. (2006) 30:344–60. 10.1007/s11031-006-9051-8

[35] Bormann D Greitemeyer T. Immersed in virtual worlds and minds: effects of in-game storytelling on immersion, need satisfaction, and affective theory of mind. Soc Psychol Pers Sci. (2015) 61:646–52. 10.1177/1948550615578177

[36] Mar RA Oatley K. The function of fiction is the abstraction and simulation of social experience. Perspect Psychol Sci. (2008) 3:173–92. 10.1111/j.1745-6924.2008.00073.x 26158934

[37] Bopp JA Müller LJ Aeschbach LF Opwis K Mekler ED. Exploring emotional attachment to game characters. In: Proceedings of the Annual Symposium on Computer-Human Interaction in Play. Barcelona: Association for Computing Machinery (ACM) (2019). p. 313–24. 10.1145/3311350.3347169

[38] Rigby CS Ryan RM. Motivation for entertainment media and its eudaimonic aspects through the lens of self-determination theory. In: The Routledge Handbook of Media Use and Well-being: International Perspectives on Theory and Research on Positive Media Effects. New York, NY: Routledge (2016). p. 34–48.

[39] Allen JJ Anderson CA. Satisfaction and frustration of basic psychological needs in the real world and in video games predict internet gaming disorder scores and well-being. Comput Hum Behav. (2018) 84:220–9. 10.1016/j.chb.2018.02.034

[40] Rigby S Ryan RM. Glued to Games: How Video Games Draw Us in and Hold Us Spellbound: How Video Games Draw Us in and Hold Us Spellbound (Santa Barbara, CA: AbC-CLIo) (2011).

[41] Wan CS Chiou WB. Why are adolescents addicted to online gaming? an interview study in taiwan. Cyberpsychol Behav. (2006) 9:762–6. 10.1089/cpb.2006.9.762 17201603

[42] Melodia F Canale N Griffiths MD. the role of avoidance coping and escape motives in problematic online gaming: a systematic literature review. Int J Mental Health Addict. (2020) 1–27. 10.1007/s11469-020-00422-w

[43] Blasi MD Giardina A Giordano C Coco GL Tosto C Billieux J. Problematic video game use as an emotional coping strategy: evidence from a sample of mmorpg gamers. J Behav Addict. (2019) 8:25–34. 10.1556/2006.8.2019.02 30739460 PMC7044601

[44] van den Eijnden R Koning I Doornwaard S van Gurp F ter Bogt T. The impact of heavy and disordered use of games and social media on adolescents' psychological, social, and school functioning. J Behav Addict. (2018) 7:697–706. 10.1556/2006.7.2018.65 30264607 PMC6426368

[45] Teng Z Pontes HM Nie Q Xiang G Griffiths MD Guo C. Internet gaming disorder and psychosocial well-being: a longitudinal study of older-aged adolescents and emerging adults. Addict Behav. (2020) 110:106530. 10.1016/j.addbeh.2020.106530 32683173

[46] Eren HK Örsal Ö. Computer game addiction and loneliness in children. Iran J Public Health. (2018) 47:1504–10. 30524980 PMC6277725

[47] Nap-van der Vlist MM Dalmeijer GW Grootenhuis MA van der Ent CK van den Heuvel-Eibrink MM Wulffraat NM. Fatigue in childhood chronic disease. Arch Dis Childh. (2019) 104:1090–5. 10.1136/archdischild-2019-316782 31175124

[48] Pao M Bosk A. Anxiety in medically ill children/adolescents. Depress Anxiety. (2011) 28:40–9. 10.1002/da.20727 20721908 PMC2990785

[49] Greenham M Hearps S Gomes A Rinehart N Gonzalez L Gordon A. Environmental contributions to social and mental health outcomes following pediatric stroke. Dev Neuropsychol. (2015) 40:348–62. 10.1080/87565641.2015.1095191 26491988

[50] Berkelbach van der Sprenkel EE Nijhof SL Dalmeijer GW Onland-Moret NC de Roos SA Lesscher H. Psychosocial functioning in adolescents growing up with chronic disease: the Dutch HBSC study. Eur J Pediatr. Berlin (2022) 181:763–73. 10.1007/s00431-021-04268-9 34595612 PMC8821406

[51] Stevens GWJM van Dorsselaer S Boer M de Roos S Duinhof E ter Bogt T. HBSC 2017. Gezondheid en welzijn van jongeren in Nederland. (Utrecht University) (2018).

[52] [Dataset] Nijhof S Putte Evd Hoefnagels JW. Proactive cohort study (2021). 10.34894/FXUGHW

[53] Nap-van der Vlist MM Hoefnagels JW Dalmeijer GW Moopen N van der Ent CK Swart JF. The proactive cohort study: rationale, design, and study procedures. Eur J Epidemiol. (2022) 37:993–1002. 10.1007/s10654-022-00889-y 35980506 PMC9385417

[54] Goedhart A Treffers PD Van Widenfelt B. Vragen naar psychische problemen bij kinderen en adolescenten. de strengths and difficulties questionnaire (sdq). Maandblad geestelijke volksgezondheid (2003) 58:1018–35.

[55] Goodman R Meltzer H Bailey V. The strengths and difficulties questionnaire: a pilot study on the validity of the self-report version. Eur Child Adolesc Psychiatry. (1998) 7:25–130. 9826298 10.1007/s007870050057

[56] Muris P Meesters C van den Berg F. The strengths and difficulties questionnaire (sdq). Eur Child Adolesc Psychiatry. (2003) 12:1–8. 10.1007/s00787-003-0298-2 12601558

[57] Gadermann AM Guhn M Zumbo BD. Estimating ordinal reliability for likert-type and ordinal item response data: a conceptual, empirical, and practical guide. Pract Assess Res Eval. (2012) 17:3.

[58] Duinhof EL Stevens GWJM Van Dorsselaer S Monshouwer K Vollebergh WA. Ten-year trends in adolescents' self-reported emotional and behavioral problems in the netherlands. Eur Child Adolesc Psychiatry. (2015) 24:1119–28. 10.1007/s00787-014-0664-2 25534927

[59] Lemmens JS Valkenburg PM Gentile DA. The internet gaming disorder scale. Psychol Assess. (2015) 27:567. 10.1037/pas0000062 25558970

[60] Lawrence C. Take a load off: cognitive considerations for game design. In: Proceedings of the 3rd Australasian Conference on Interactive Entertainment. Perth, WA: ACM International Conference Proceeding Series (2006). p. 91–5.

[61] van der Rijst VG van der Neut D Peeters M. Teaming up to battle against problematic gaming behavior: the relationship between social vulnerabilities and (problematic) game play using 3 different perspectives (n.d.). Unpublished at the time of writing.

[62] Verheijen GP Burk WJ Stoltz SE van den Berg YH Cillessen AH. Associations between different aspects of video game play behavior and adolescent adjustment. J Media Psychol Theor Methods Applic. (2020) 32:27. 10.1027/1864-1105/a00025329580195

[63] Verheijen G. The interplay between video games and social adjustment in adolescence. (Ph.D. thesis) Radboud University, Nijmegen. Sl: sn (2020).29174875

